# An Address to the Kensington Branch of the League of Nations Union

**Published:** 1921-05-07

**Authors:** William J. Collins


### SOME INTERNATIONAL HEALTH QUESTIONS AND THE LEAGUE OF NATIONS.

### An Address to the Kensington Branch of the League of Nations Union.

By Sir WILLIAM J. COLLINS, K.C.V.O., M.S., M.D., B.Sc. (Lond.), F.R.C.S. (Eng.).

Typhus is the touchstone of sanitary neglect, the fruit and nemesis of mal-hygiene. Its propagation is now largely attributed to pediculi, and although bacteriology has failed to identify a micro-organism with its causation, no disease is more amenable to a sanitary environment and the vigorous application of the gospel of soap and water. In the memorable words of King Edward, "If preventable, why not prevented?" and it urgently behoves the wealthier nations of the world to co-operate in stamping out this *morbus miseriæ* in Eastern Europe, if not on altruistic, then at the lowest on prudential grounds, as precaution against the extension of the infection to themselves.

### THE INTERNATIONAL HEALTH OFFICE.

In regard to the institution of a "Permanent International Health Office" the Council of the League on February 15 and March 13, 1920, passed resolutions suggesting a Conference in London to prepare proposals for a Permanent body to advise on questions connected with the execution of Articles 23 (*f*) and 25. The Conference, which was held at the Ministry of Health in April 1920, comprised representatives of France, Great Britain, Italy, Japan, and the United States, and was also attended by nominees of the League of Red Cross Societies and of the Secretariate of the League of Nations as well as the nominee of the *Office International d'Hygiène Publique* in Paris.

Here, as in other cases, such as that of the Red Cross Societies, the new *post-bellum* activities found the ground in part preoccupied by *ante-bellum* organisations, and the danger and complications of overlapping jurisdiction in the international field became obvious.

The *Office International d'Hygiène* was the outcome of sanitary conventions drafted in Paris and Rome in 1903 and 1907, whereby a bureau was established in Paris, with a Director and Secretary, for effecting communications between the health authorities of the signatory Powers and for the periodical issue of bulletins and statistics in regard to infectious diseases.

Primarily concerned with plague, cholera, and yellow fever, its scope has been extended to other epidemics, and, although several Powers have not acceded to the Rome Convention, the Paris bureau has become a useful nucleus of international epidemiological information.

Plague from the mediæval times has led the Western nations to institute lazarettos and quarantine precautions, and, though practically absent from this country since the seventeenth century, its menace in 1720 led the learned Dr. Mead to lay down rules for combating its contagion, which marked a great advance in sanitary administration. It was not till 1831 that Asiatic cholera, so-called, made its first serious visitation in Europe, and led to the great reforms in water supply of our towns which have been so markedly beneficial. Yellow fever, a tropical and sub-tropical endemic, has been traced to conveyance by mosquitos, and, by steps directed against the latter and their breeding-places, wonderful results, associated with the name of the late Colonel Gorgas, have been recorded in Panama and elsewhere.

The scheme which I understand was laid before the assembly of the League had for its object the merging of the existing *Office d'Hygiène* in Paris in the new international health organisation of the League, with headquarters at the seat of the League at Geneva, with a medical secretary and staff. The new constitution reflects that of the Council of the League as regards its General Committee, and the *Office d'Hygiène* as regards its Executive; while its functions are:

(1) To secure a quicker and more effective transmission of notification of the outbreak and distribution of epidemic diseases.

(2) To provide a more rapid method of revising and preparing international sanitary Conventions.

(3) To secure greater uniformity in the collection of health statistics and in the nomenclature of diseases.

It is further authorised to advise the Labour Section of the League on questions of health, and to confer with the League of Red Cross Societies when requested to do so.

Representatives of the latter League and of Labour are to be placed on its Executive Committee.

The International Labour Conference, which met at Washington in November 1919, approved of six draft conventions which, when effectuated, should have important influence in regard to industrial hygiene. These Conventions dealt with an eight-hours day, the exclusion from industries of children under fourteen, women's non-employment between 10 P.M. and 5 A.M. and for six weeks after child-birth, and the non-employment of persons under eighteen between 10 P.M. and 5 A.M.

### THE RED CROSS MOVEMENT.

Inasmuch as the work of national Red Cross organisations is the subject of a special article (No. 25) in the Covenant, and as the recently constituted League of those bodies is accorded representation on the new international health authority, I must briefly allude to that great chapter in the history of international humanity and philanthropy.

This is not the occasion to relate at length the story of the Red Cross movement. To Henri Dunant, of Geneva, is justly ascribed the origination of that reform, although I believe the Italians have disputed his priority. Having witnessed the awful neglect and suffering of the wounded on the stricken fields of Magenta and Solferino in 1859, he published in 1862 his "Souvenir de Solferino," describing his experiences. When M. Dunant visited London in 1872 he chivalrously said: "Though I am known as the founder of the Red Cross and the originator of the Convention of Geneva, it is to an English woman that all the honour of that Convention is due. What inspired me to go to Italy during the war of 1859 was the work of Miss Florence Nightingale in the Crimea."

I had, in the earlier years of this century, several privileged conversations with Miss Nightingale, and, notwithstanding the detractions of a recent biographer, I retain my admiration derived from personal knowledge of that great lady. It is true that the "lady with the lamp" could be the lady with the lash when exposing official incompetence in high places, but a wiser, saner, or more liberal-minded counsellor it has seldom been my privilege to meet in either sex.

A Swiss Society, "La Société Genevoise d'Utilité Publique," took up the cause and led to the assembly of a Diplomatic Conference at Geneva in 1864, which drafted the Convention to which Great Britain acceded the following year. The Convention provided for the neutralisation of ambulances, hospitals, staff, and stores devoted to the succour of the sick and wounded in warfare, under the distinctive Red Cross flag. In the room in which the Conference was held, and the memorable Convention was signed, you will find interesting allegories and associations. There you may see, in actuality, what we have read of in prophecy—ploughshares made out of swords, and spears converted into pruning-hooks; while the President's bell, which calls international deliberations to order, is cast from cannon whose foul mouth formerly belched out destruction and death.

In that room, too, as a plaque on the wall reminds one,

(Continued from page 82)

**Some International Health Questions**—(*continued*).

the Alabama claim was settled, albeit against ourselves; but the fact of such arbitraments between us and America paved the way for arbitration treaties and tribunals to which, despite the bitter experience of the last six years, we still look as the alternatives to appeals to the sword.

Dunant's labours did not escape criticism from the "letting things alone" party, and official militarism was voiced by Maréchal Randon, who exclaimed, "Why should these civilians mix themselves up with matters which do not concern them?" and resented the intervention of voluntary aid societies in the sphere of military operations of belligerent nations. Nevertheless, nearly every Power came in time to accede to the Convention, and in 1906 another Geneva Conference was held (attended by representatives of thirty-seven Powers), at the instance of the first Hague Peace Conference of 1899. The object of this Conference, which formulated the Geneva Convention of 1906, was to improve on the terminology and extend the provisions of the Convention of 1864, and of a revision thereof which had been attempted in 1868. The Convention of 1906 operates as between those Powers which have ratified it, while that of 1864 remains in force between those Powers which signed it, but which have not ratified or adhered to the later Convention.

Notwithstanding the efforts of Longmore and Brackenbury, and the inauguration on the Continent of many *Sociétés de secours aux militaires blessés*, no similar organisations took shape in this country until 1870, when a "National Society for the Aid of the Sick and Wounded in War" was started, with Lord Wantage as President. In 1905 this Society fused with the Central British Red Cross Council, and was incorporated by Royal Charter in 1908 under the name of "The British Red Cross Society," with its primary object "furnishing aid to the sick and wounded in time of war," its operations to be "under the direct supervision and control, and with the consent of, the military authorities." As Lord Wantage truly observed, "However well organised an Army Medical Service may be, it never has been, and never will be, able to cope adequately with sudden emergencies of war in a large scale; *voluntary* organisations, unimpeded by official restrictions, are alone capable of giving auxiliary relief and of providing extra comforts and luxuries with the requisite promptitude and rapidity." What the Joint Committee of the British Red Cross Society and the Order of St. John developed into during the Great War is fresh in the memory of us all. More than 16 million pounds were collected and distributed, and its beneficent influence was felt in every quarter of the globe. In this case, as in others, not only did the State not shoulder the burden of the voluntary agencies, but the voluntary agencies shouldered the burden of the State. When the war was over the Society obtained an Act of Parliament to enable it to use its surplus funds for the relief of sickness, suffering, or distress in the British Empire or the countries of the Allies; and more recently it petitioned for, and obtained, a new and supplemental charter to enable it, in addition to its primary object, to include within its powers those set out in Article 25 of the Covenant of the League of Nations, viz.: "The improvement of health, the prevention of disease, and the mitigation of suffering throughout the world."

The close of the Great War in other countries besides our own found Red Cross Societies confronted with the problem of either demobilising and dispersing the great organisations they had evolved, or else preserving them and diverting their activities into other beneficent channels. A committee of Red Cross Societies, comprising representatives from the United States, Great Britain, France, Italy, and Japan, was accordingly assembled at Cannes, at the instance of the American Society, and agreed on combining to continue. They consulted with the "International Committee of the Red Cross" at Geneva, which had existed for more than fifty years, and of which my friend Dr. D'Espine is, I believe, still Vice-President; but it was agreed that its scope and organisation, well adapted for times of war, were hardly suitable for the new peace objects in contemplation. The Committee of the five national Red Cross Societies accordingly summoned a medical conference at Cannes in April 1919. An elaborate and ambitious programme was unfolded by Colonel Strong, of the American delegation, with a central bureau of hygiene, a large staff, fourteen divisions, laboratories, and museum, and the objective was stated to be "to determine how the message which science has to give can best be presented to the world to improve the health, happiness, and general well-being of mankind." Seven sections were appointed to report respectively on child welfare, tuberculosis, venereal diseases, malaria, nursing, preventive medicine, and public education, etc., and resolutions arising out of these reports were duly adopted by the Conference as a whole.

The Conference being constituted by the association of national voluntary agencies, partook largely of the nature of medical and scientific congresses, with which we have been familiar in the past, for the comparison and exchange of experience between researchers and administrators in different nations. While its deliberations, though extensive, did not cover the whole range of sanitary science or hygiene, they went considerably beyond the more essentially international health questions, such as can only be effectually tackled by co-ordinated international co-operation. Indeed, just as the Conference was about to rise from its labours, a telegram was received describing "the alarming conditions existing in Central Europe by reason of the outbreak and spread of typhus fever." That was on April 10, 1919. Nevertheless, the effective grappling with that problem is still demanding the practical intervention, not only of the official bureaux, but also of voluntary aid associations, with their greater elasticity and more rapid mobility in operation. The League of Red Cross Societies, with articles of association, was organised in Paris shortly after the Cannes Conference, and the first meeting of its General Council, at which twenty-seven nationalities were represented (as well as the League of Nations and the Red Cross International Committee), was held at Geneva in March 1920, when its objects were promulgated in resolutions, and a relationship established with the *Office International d'Hygiène* in Paris.

There is another health question which has engaged, is engaging, and will continue to engage, the attention of the League of Nations, and one with which I have had some concern, viz., the international control of traffic in dangerous drugs—so-called habit-forming drugs or drugs of addiction, such as opium, morphia, cocaine, and their derivatives. I was one of the British representatives at the three International Opium Conferences held at the Hague in 1911, 1913, and 1914, which drafted, and provided for putting in force, the Opium Convention of 1912. At the close of the third Conference, just before the outbreak of war, forty-two out of forty-six Powers had *signed* the Convention, eleven had *ratified* it, and fourteen more were ready to do so, while a special protocol was opened for signature by those Powers which were ready to enforce it without waiting for the rest. A Commission which sat in Shanghai in 1909 realised that the question was not one affecting the Orient alone, but that the Occident also has its morphia-maniacs, its cocaine-adicts, and its opium dens. Any legislative or administrative effort to check the abuse, it is clear, must be international in its scope and directed to the source and production, as well as to the distribution, of these dangerous drugs.

It is idle to stop the Indian export to China if China raises an abundant harvest of the poppy or imports morphia by the ton from Britain, or from Japan and the United States. The abuse of these drugs, beneficent though their medical usage be, is world-wide, and international action can alone stand a chance of success. The Convention which we drafted in 1912 accordingly provided for stringent regulation of the manufacture, import, sale, distribution, and

(*Continued on p.* 106.)

### Some International Health Questions and the League of Nations.

(*Continued from page* 100.)

export of these drugs, and the restriction of preparations containing more than one-fifth per cent. of the alkaloids to medical and legitimate purposes only.

The problem now presented is to secure effectuation of the Treaty by *all* the Powers and the passage and putting into force of the requisite legislation and ordinances. The treaties with the late enemy Powers have required their adhesion (which had been previously withheld), and all the signatories at Versailles bound themselves to enact the necessary legislation within a twelvemonth of the Treaty coming into force. The Dangerous Drugs Act passed by our Parliament in July 1920 may serve as a model for such legislation.

The Netherlands Government, which, in accordance with the Articles of the Opium Convention, has hitherto most courteously and efficiently acted as an intermediary between the different Powers in effectuating its operation, submitted a memorandum to the Assembly of the League at Geneva. This memorandum invited the consideration by the Assembly, in view of Article 23 (*f*) of the Covenant, of the next steps which should be taken by the League to secure the universal carrying out of the restrictions on the traffic in these noxious drugs, and called attention to the fact that the Powers which have ratified the Convention are not the same as those which are members of the League.

There is need of the "international mind," as well as the humanitarian heart, in dealing with all these questions on which I have lightly touched, whether it be the care of native races, the suppression of slavery, the war against epidemic disease, solicitude for the sick and wounded, or the control of traffic in noxious drugs. The solidarity of mankind makes the need for united action self-evident, for—

"Mankind is one by nature, and an impulse bears along,
Round the earth's electric circle, the swift flash of right or wrong;
Whether conscious or unconscious, yet humanity's vast frame
Thro' its ocean-sunder'd fibres feels the throb of joy or shame;
In the gain or loss of one race all the rest have equal claim."

While, then, neither seeking to overload the new-born League with duties which may well be dealt with nationally or municipally, nor subordinating voluntary agencies to bureaucratic thraldom, there is room and occasion for re-adjusting and revising international law and relationships from the standpoint of health and the prevention of disease, remembering that—

"New occasions teach new duties;
Time makes ancient laws uncouth.
They must upward be and onward,
Who would keep abreast of Truth."

